# Preclinical assessment of galunisertib (LY2157299 monohydrate), a first-in-class transforming growth factor-β receptor type I inhibitor

**DOI:** 10.18632/oncotarget.23795

**Published:** 2017-12-31

**Authors:** Jonathan M. Yingling, William T. McMillen, Lei Yan, Huocong Huang, J. Scott Sawyer, Jeremy Graff, David K. Clawson, Karen S. Britt, Bryan D. Anderson, Douglas W. Beight, Durisala Desaiah, Michael M. Lahn, Karim A. Benhadji, Maria J. Lallena, Rikke B. Holmgaard, Xiaohong Xu, Faming Zhang, Jason R. Manro, Philip W. Iversen, Chandrasekar V. Iyer, Rolf A. Brekken, Michael D. Kalos, Kyla E. Driscoll

**Affiliations:** ^1^ Idera Pharmaceuticals, Inc., Cambridge, Massachusetts, USA; ^2^ Lilly Research Laboratories, Eli Lilly and Company, Indianapolis, IN and New York, NY, USA; ^3^ Hamon Center for Therapeutic Oncology Research, University of Texas Southwestern Medical Center, Dallas, TX, USA

**Keywords:** TGFβ receptor I, SMAD, galunisertib, LY2157299

## Abstract

Transforming growth factor-β (TGFβ) is an important driver of tumor growth via intrinsic and extrinsic mechanisms, and is therefore an attractive target for developing cancer therapeutics. Using preclinical models, we characterized the anti-tumor activity of a small molecule inhibitor of TGFβ receptor I (TGFβRI), galunisertib (LY2157299 monohydrate). Galunisertib demonstrated potent and selective inhibition of TGFβRI with corresponding inhibition of downstream signaling via inhibition of SMAD phosphorylation (pSMAD). Galunisertib also inhibited TGFβ-induced pSMAD in vivo, which enabled a pharmacokinetic/pharmacodynamic profile in Calu6 and EMT6-LM2 tumors. Galunisertib demonstrated anti-tumor activity including inhibition of tumor cell migration and mesenchymal phenotype, reversal of TGFβ-mediated immune-suppression, and tumor growth delay. A concentration-effect relationship was established with a dosing schedule to achieve the optimal level of target modulation. Finally, a rat model demonstrated a correlation between galunisertib-dependent inhibition of pSMAD in tumor tissues and in PBMCs, supporting the use of PBMCs for assessing pharmacodynamic effects.

Galunisertib has been tested in several clinical studies with evidence of anti-tumor activity observed in subsets of patients. Here, we demonstrate that galunisertib inhibits a number of TGFβ-dependent functions leading to anti-tumor activity. The enhanced understanding of galunisertib provides rationale for further informed clinical development of TGFβ pathway inhibitors.

## INTRODUCTION

The transforming growth factor beta (TGFβ) superfamily is an important group of signaling receptors triggered by a broad set of ligands that are critical for development and tissue homeostasis. The TGFβ ligand superfamily includes three TGFβ isoforms (TGFβ1, TGFβ2 and TGFβ3), activins, growth and differentiation factors, BMPs, inhibins, nodal, and anti-mullerian hormone [1–2]. The TGFβ isoforms represent prototypic members of the TGFβ superfamily and signal via a TGFβ receptor I (TGFβRI) and TGFβ receptor II (TGFβRII) heterodimeric complex; in addition to their function in normal growth and development, they also play key roles in several disease states including cancer [2–4]. The TGFβ pathway signaling has dual roles in tumor development, initially as a growth-inhibiting signal in the early stages of tumor progression but then providing tumor-promoting signals during advanced stages, inducing many activities that lead to enhanced growth, invasion, and metastasis of cancer cells [4–5]. TGFβ ligand levels are higher in many cancer patients compared to normal patients and elevated TGFβ ligand levels are observed in patients whose tumors are sensitive (i.e. receptor positive, TGFβ ligand dependent) or insensitive (i.e. receptor negative, TGFβ ligand independent) to TGFβ signaling [6–11]. Increased ligand levels are also associated with poor prognosis and severity of disease [6–8, 10–13]. Aberrant TGFβ signaling has been implicated in several human diseases, including malignancies such as glioblastoma and breast cancer [8, 10–11, 14]. Accordingly, blockade of the TGFβ pathway is an attractive anti-cancer therapeutic approach.

TGFβ plays pleiotropic roles to promote cancer through tumor cell intrinsic and extrinsic activities. Tumor cell intrinsic activities of the TGFβ pathway include autocrine TGFβ driven tumor cell proliferation and differentiation, TGFβ driven epithelial to mesenchymal transition (EMT), invasion and migration, prometastatic cytokine production and autocrine mitogen production. Tumor cell extrinsic activities include induction and increased tumor vascularization, modulation of the stromal extracellular matrix, and inhibition of immune surveillance and antitumor immunity [4, 15]. Studies in genetically engineered mouse models and preclinical studies using TGFβ pathway antagonists support the pro-metastatic function of TGFβ, although this activity may depend on multiple factors, such as the nature of the tumor-initiating mutation, the precise mechanism of TGFβ inactivation, and the timing of TGFβ signaling [4, 12–13, 16]. Small molecule TGFβRI inhibitors as well as antibodies directed to the type II receptor have been shown to block EMT and tumor cell migration in cancer cells [16–18]. In turn, TGFβ inhibition is currently being investigated as a treatment option in patients with advanced metastatic cancer [9, 19–21].

TGFβ has presented an attractive target for developing cancer therapeutics due to its multitude of critical roles in progression and metastasis in advanced cancers. However, the pleiotropic roles of TGFβ signaling in normal cell development, the complexity of the TGFβ superfamily signaling, and the structural overlap of TGFβ receptor signaling domains with other signaling molecules have complicated the effective clinical targeting of the TGFβ pathway. Nonetheless, a variety of strategies to target the TGFβ pathway have been pursued, including TGFβ pathway inhibition at the translational level using antisense oligonucleotides delivered directly into tumors, sequestering the ligand(s) with monoclonal antibodies, inhibiting TGFβ binding to the type II receptor using monoclonal antibodies, and inhibiting the receptor-mediated signaling cascade using inhibitors of TGFβ receptor kinases [21]. Several agents based on these approaches are in pre-clinical development, including those aimed to block the catalytic activity of TGFβRI that act as competitive inhibitors for the ATP-binding site of TGFβRI kinase domain. These include small molecules such as EW-7197 [22], SB-431542 and SB-505124 (GlaxoSmithKline), SD-093 and SD-208 (Scios), and LY580276 (Lilly Research Laboratories) [18, 23–25].

Using structure-based drug design and structure-activity relationships, we have identified galunisertib (LY2157299 monohydrate) as a potent and selective TGFβRI kinase inhibitor from the dihydropyrrolopyrazole class. Galunisertib is currently under clinical development for a variety of cancers [9, 19–21]. Here we describe the selectivity of galunisertib for the TGFβRI kinase domain and demonstrate the ability of galunisertib to inhibit TGFβ-dependent tumor cell intrinsic and extrinsic functions using *in vitro* and *in vivo* assays, and to inhibit tumor growth in established tumor mouse models. Finally, we demonstrated the utility of peripheral blood mononuclear cells (PBMCs) as a surrogate tissue for the assessment of pharmacodynamics and treatment effects of galunisertib, enabling informed clinical development via the potential to effectively monitor and dose the drug in patients.

## RESULTS

### Chemical structure, *in vitro* enzymatic potency, and selectivity profile of galunisertib

**Figure 1:**
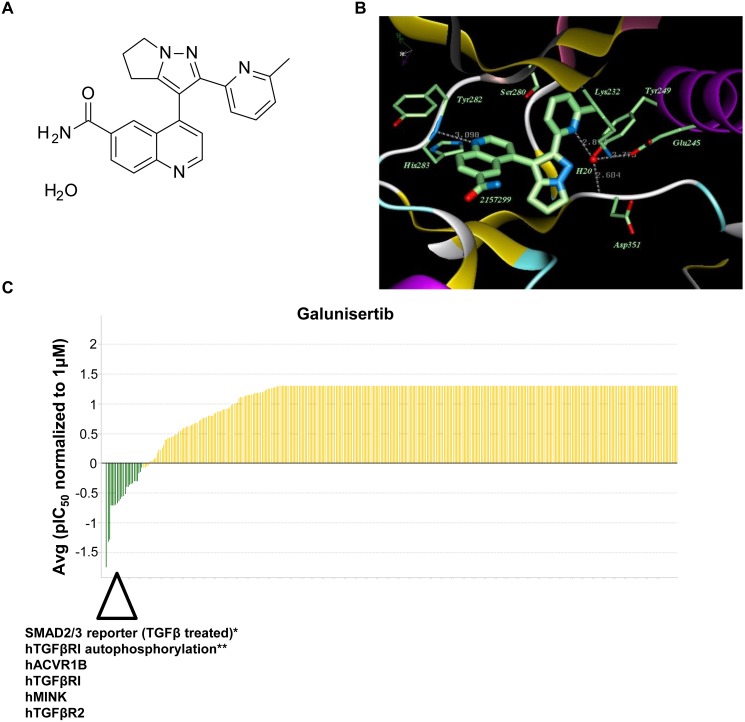
Galunisertib is a selective inhibitor of TGFβRI (Alk5) and Alk4 **A.** Chemical structure of galunisertib. **B.** X-ray co-crystal structure of galunisertib bound at the ATP-site of Alk5. Crystallography of galunisertib in the ATP-site of TGFβRI (T204D) is shown with key residues highlighted. **C.** Galunisertib selectivity profile from DiscoverX analysis of 456 kinases. Galunisertib was tested for binding activity at 3 different doses to generate a concentration response curve (CRC) from which IC_50_ values were calculated. Examples of the strongest interactions are highlighted below the waterfall plot (hACVR1B (Alk4), hTGFβRI, hMINK, hTGFβR2). *In vitro* assay data including SMAD2/3 reporter assay (TGFβ treated)* or hTGFβRI autophosphorylation assay** are included in the waterfall plot for comparison.

Galunisertib is a TGFβRI kinase inhibitor of the dihydropyrrolopyrazole class that was synthesized in a four-step convergent approach to generate a chemical compound with the formula 4-[2-(6-methylpyridin-2-yl)-5,6-dihydro-4H-pyrrolo[1,2-b]pyrazol-3-yl]quinoline-6-carboxamide monohydrate (Figure 1A) [26]. Co-crystallization of galunisertib with a recombinant TGFβRI subunit revealed that galunisertib binds to the ATP-binding site of TGFβRI (Figure 1B). The critical interaction involves an approximately 3 Å hydrogen bond between the quinoline nitrogen in galunisertib and the hinge region backbone NH hydrogen atom of histidine 283 in TGFβRI. As also shown in Figure 1B, a second key interaction involves a hydrogen bond from the pyridine nitrogen atom in galunisertib via a single ordered H_2_O molecule that anchors interactions with tyrosine 249, glutamic acid 245, and the backbone NH hydrogen atom of aspartic acid 351 in TGFβRI. Finally, also visible in the x-ray co-crystal structure is the interaction of galunisertib with the gatekeeper residue serine 280, which has been implicated in the Alk5/Alk2 selectivity profile observed with other structurally related inhibitors [27].

The selectivity and potency of galunisertib for TGFβRI was assessed using the KINOMEscan platform (DiscoverX), which measures interactions of test compounds against a panel of 456 wild-type and disease-relevant mutant kinases. To enable direct comparison of IC_50_ data across kinases, these assays were run in the absence of ATP and using a three-point titration of galunisertib. These analyses showed that galunisertib is a highly selective TGFβRI inhibitor with an IC_50_ for the TGFβRI/Alk5 kinase domain of 0.172 μM (Figure 1C, Table 1). Sub-micromolar IC_50_ values were observed for a very select number of related kinases including TGFβRII (0.21 μM) and Alk4/ACVR1B (0.08 μM, a closely related TGFβ superfamily member at the kinase domain level [2]). More modest inhibition was observed for ACVR2B (0.69 μM) and Alk6/BMPR1B (0.47 μM), receptors involved in activin and BMP signaling. Table 1 shows a list of sub-micromolar interactions with IC_50_ values. Finally, very low activity was observed against Alk2/ACVR1 (35.7 μM), ACVR2A (35.7 μM), Alk1/ACVRL1 (24.9 μM), Alk3/BMPR1A (16.8 μM), and BMPR2 (>60.0 μM) receptors.

**Table 1:** Summary of the DiscoverX selectivity profile of galunisertib

Enzyme/Receptor	Galunisertib IC_50_ (μM)
**TGFβR1 (Alk5)**	**0.172**
**Alk4/ACVR1B**	0.0777
**MINK**	0.19
**TGFβR2**	0.208
**RIPK2**	0.22
**CSNK1A1**	0.26
**MAP4K4**	0.28
**GAK**	0.31
**CSNK1E1**	0.4
**Alk6/BMPR1**	0.471
**Braf**	0.5
**TNIK**	0.51
**ACVR2B**	0.694
**RSK4**	0.72
**Abl1**	0.86
**ZAK**	0.86
**NLK**	0.91
**Alk3/BMPR1A**	16.8
**Alk1/ACVRL1**	24.9
**Alk2/ACVR1**	35.7
**ACVR2A**	35.7
**BMPR2**	>60.0

Galunisertib demonstrated a Ki of 86 nM with an IC_50_ of 0.051 nM in autophosphorylation kinase assays (Table 2). The constitutively active T204D mutant was used in these assays to enhance sensitivity. Activity of galunisertib in autophosphorylation assays testing TGFβRII showed an IC_50_ of 2 μM (Table 2). Confirmatory binding assays done to test galunisertib binding to TGFβRII showed an IC_50_ of 0.43μM, in agreement with the DiscoverX profiling assay (Table 2). Finally, analysis in the CEREP diversity panel demonstrated <25% inhibition by galunisertib at 10 μM against all 65 targets (data not shown).

**Table 2:** Summary of *in vitro* enzymatic activities of galunisertib

Target	Assay	Result
**TGFβR1**	Ki	86 nM
**TGFβRI T204D**	Autophosphorylation kinase assay	IC_50_: 0.05 μM
**TGFβRII**	Autophosphorylation kinase assay	IC_50_: 2 μM
**TGFβRII**	Binding assay	IC_50_: 0.43 μM
**TGFβ ligand**	Cell based reporter assay (SMAD2/3)	IC_50_: 221 nM
**BMP ligand**	Cell based reporter assay (SMAD1/5/8)	IC_50_: >10000 nM
**4T1-LP**	pSMAD activity	IC_50_: 1.77 μM
**EMT6-LM2**	pSMAD activity	IC_50_: 0.89 μM

### Selective inhibition of TGFβ-induced SMAD phosphorylation and cell proliferation

**Figure 2:**
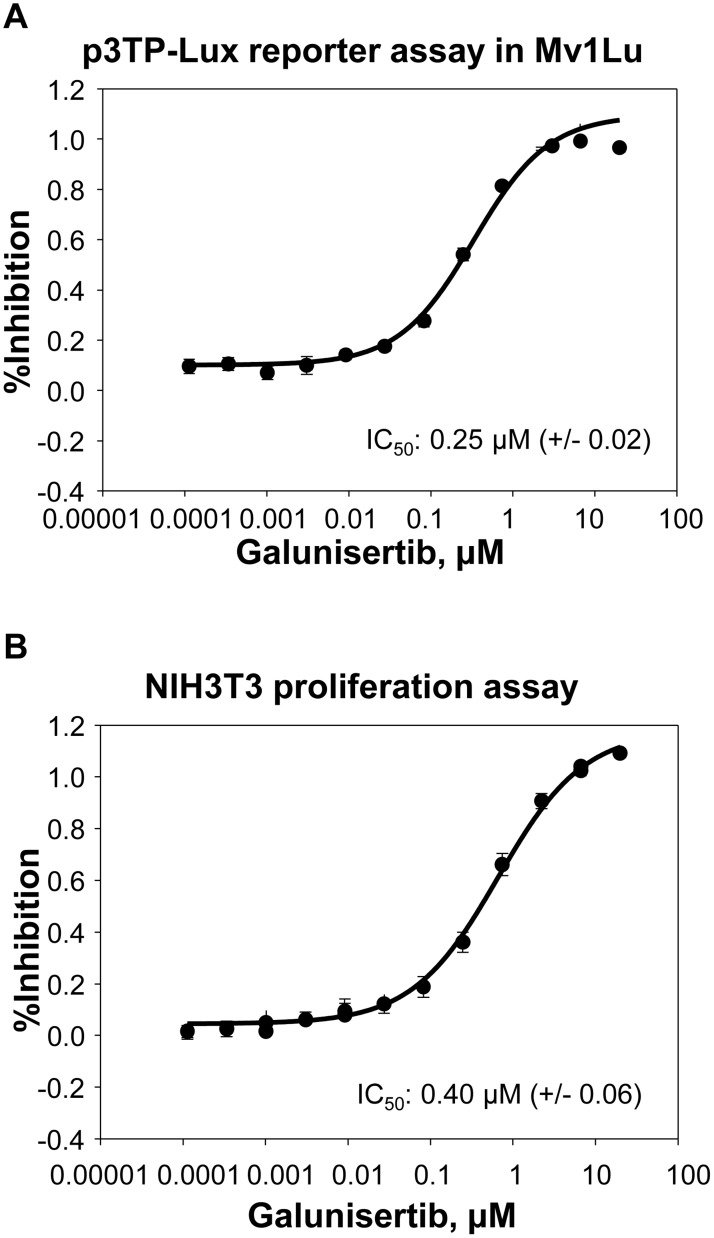
Galunisertib inhibits TGFβ mediated signaling Galunisertib was tested for the ability to inhibit activity in the p3TP-Lux reporter cell line **A.** or inhibit proliferation in NIH3T3 fibroblasts **B.** Increasing concentrations of galunisertib were tested for the ability to inhibit activity from the respective cell lines. The assay was run 4 times, each with 3-8 replicates per experiment. A representative dose/response curve is shown for each assay.

The ability of galunisertib to inhibit TGFβ-mediated signaling was assessed in Mv1Lu reporter gene assays, growth factor-induced NIH3T3 proliferation assays, and in-vitro assays to directly measure pSMAD inhibition in tumor cell lines. SMAD2/3 phosphorylation is an immediate downstream signaling event following ligand (TGFβ1, 2, or 3) binding to the type II receptor (TGFβRII)/type I receptor (TGFβRI/Alk5) complex [2, 28]. Activated SMAD complexes containing SMAD2 or SMAD3 bind SMAD4 and translocate to the nucleus where they regulate the expression of downstream genes [29–30]. A summary of the IC_50_ values for each of these assays is presented in Table 2. In cell-based luciferase reporter assays, galunisertib inhibited luciferase reporter activity from TGFβ-stimulated HEK293_SMAD2/3 cells with an IC_50_ value of 221 nM and an IC_50_ value of >10000 nM for HEK293_SMAD1/5/8, supporting its highly selective profile to inhibit the canonical TGFβ signaling pathway but spare the related SMAD1/5/8 pathway (Table 2). Galunisertib also inhibited TGFβ1-mediated luciferase production from the TGFβ-responsive p3TP-Lux reporter in Mv1Lu cells with an IC_50_ of 0.251 μM (representative curve shown in Figure 2A, Table 3). In TGFβ1 stimulated Mv1Lu cells, galunisertib inhibited endogenous pSMAD with an IC_50_ = 0.176 μM, confirming direct modulation of the TGFβ signaling pathway in this cell line (data not shown).

**Table 3:** Summary of in vitro cell-based activities of galunisertib

Cell-based assay	Galunisertib
	IC_50_ (SE) μM
**Mv1Lu p3TP-Lux**	0.25 (0.02)
**Mv1Lu proliferation**	0.56 (0.11)
**NIH3T3 proliferation:** TGF-β-stimulated	0.40 (0.06)
**NIH3T3 proliferation:** PDGF-stimulated	>20
**NIH3T3 proliferation:** bFGF-stimulated	>20

Abbreviations: IC_50_ = Inhibitory concentration at 50%; SE = standard error. Assays were carried out a minimum of three times

**Figure 3:**
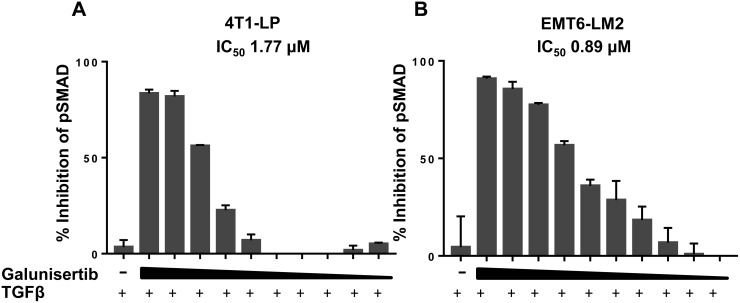
Galunisertib inhibits TGFβ mediated cellular SMAD phosphorylation *in vitro* Galunisertib inhibits TGFβ-induced pSMAD in 4T1-LP (**A**) or EMT6-LM2 (**B**) cells *in vitro*. Cells were treated with increasing concentrations of galunisertib (0.20 - 10 μM) then stimulated with TGFβ1 ligand. Following incubation, p- and tSMAD levels were assessed by ELISA. % inhibition of pSMAD2 was calculated as a function of TGFβ1 alone treated cells.

In cell modulatory assays testing NIH3T3 fibroblasts, galunisertib inhibited TGFβ1 induced proliferation with an IC_50_ of 0.396 μM (Figure 2B, Table 3). This assay represents one of the target tissues in tumors, namely stromal fibroblasts, which are pro-tumorigenic. Direct analysis of the immediate downstream target of TGFβRI confirmed functional activity for galunisertib with an IC_50_ = 0.064 μM for inhibition of pSMAD in NIH3T3 cells (data not shown); however, galunisertib did not prevent bFGF or PDGF stimulated proliferation of NIH3T3 cells (Table 3), showing specificity of the compound for TGFβ signaling modulation. Finally, galunisertib inhibited TGFβ1-induced pSMAD in the murine triple negative breast tumor cell lines 4T1-LP (Figure 3A) and EMT6-LM2 (Figure 3B) in a dose dependent manner with IC_50_ of 1.765 μM and 0.8941 μM, respectively. Thus, galunisertib potently and selectively inhibits TGFβ dependent signal transduction events in normal and tumor cell lines with functional *in vitro* pharmacology effects expected for a selective TGFβRI inhibitor.

### Effect of galunisertib on TGFβ dependent cellular pSMAD *in vivo*

**Figure 4:**
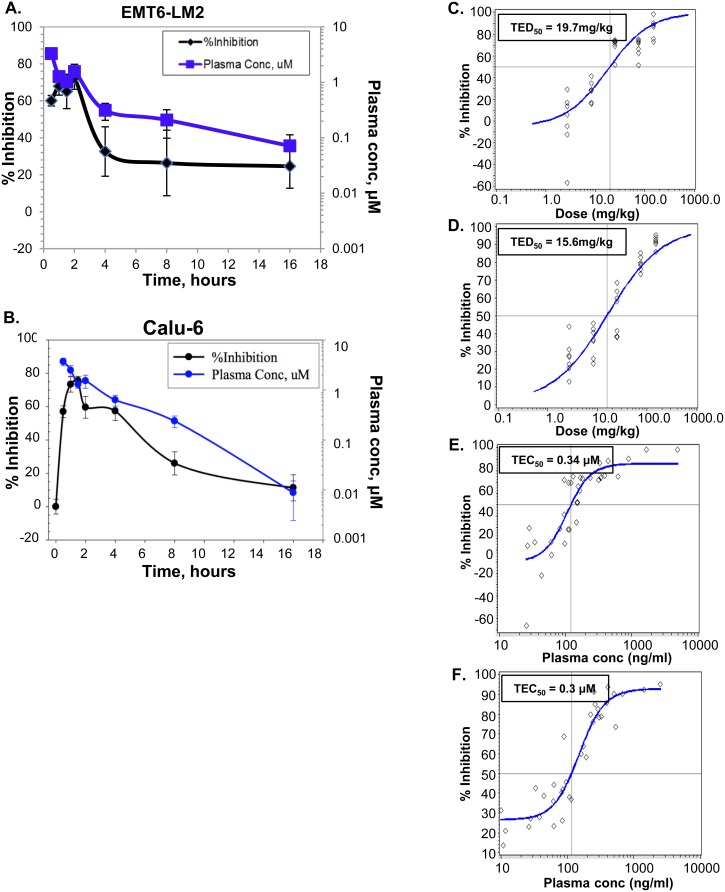
*In vivo* potency and target inhibition by galunisertib in EMT6 and Calu6 tumor models **A.**, **B.** Time-course study was performed in the syngenic EMT6-LM2 (A) or human xenograft Calu6 (B) tumor model. A single 25-mg/kg dose of galunisertib was administered to mice by oral gavage and inhibition of pSMAD levels in tumors or plasma exposures were measured at the indicated time points. **C.**, **D.** Galunisertib was administered orally to EMT6-LM2 (C) and Calu6 (D) tumor-bearing animals with a single dose of 2.7, 8.3, 25, 75 and 150 mg/kg. The inhibition of pSMAD in tumors as a function of dose is presented and the total effective dose to inhibit 50% pSMAD (TED_50_) was calculated. **E.**, **F.** Compound levels in plasma were measured following a single dose as above and the exposure-response in EMT6-LM2 (E) or Calu6 (F) was determined. Total effective concentration to inhibit 50% pSMAD (TEC_50_) was calculated for each tumor model.

The ability of galunisertib to inhibit endogenous TGFβ-dependent signal transduction *in vivo* was evaluated in Calu6 human xenografts and EMT6-LM2 murine syngenic tumor models. Time- and dose-dependent kinetic analysis of galunisertib-mediated inhibition of pSMAD and plasma concentrations of galunisertib was determined after oral administration in each tumor model. These analyses demonstrated a galunisertib time (Figure 4A, 4B) and dose (Figure 4C, 4D) -dependent inhibition of pSMAD in both models. The total effective doses corresponding to 50% inhibition (TED_50_, 95% CI) for galunisertib were estimated to be 19.7 mg/kg and 15.6 mg/kg (Figure 4C and 4D), and the total effective concentration corresponding to 50% inhibition (TEC_50_, 95% CI) were 0.34 μM and 0.3 μM (Figure 4E and Figure 4F) for the EMT6-LM2 and Calu6 models, respectively. The dose response analysis of galunisertib resulted in an estimate of 3-6 μM for maximal target inhibition *in vivo*. This was the C_max_ target plasma concentration in the time course experiments and was achieved with a 75-mg/kg dose, the TED_90_ (data not shown). In both models, maximum plasma exposure of up to 6 μM were achieved by 30 minutes (t_max_), which corresponded to ~70% pSMAD inhibition [31]; since the half-life of pSMAD is 30 minutes [30], the observed pSMAD inhibition suggests 100% inhibition of kinase activity at those plasma concentrations (Figure 4A, 4B). Thereafter, the plasma concentration of the inhibitor decreased, resulting in a gradual increase in the TGFβRI kinase activity reflected in increased pSMAD. The PK/PD profile of galunisertib demonstrates ≥20% of target inhibition *in vivo* over 12 hours following a single oral dose with an exposure of 3-5 μM, and suggests that oral administration at 75 mg/kg twice daily, can achieve significant target modulation *in vivo* over a 24-hour period.

### Effect of galunisertib on EMT and migration *in vitro*

**Figure 5:**
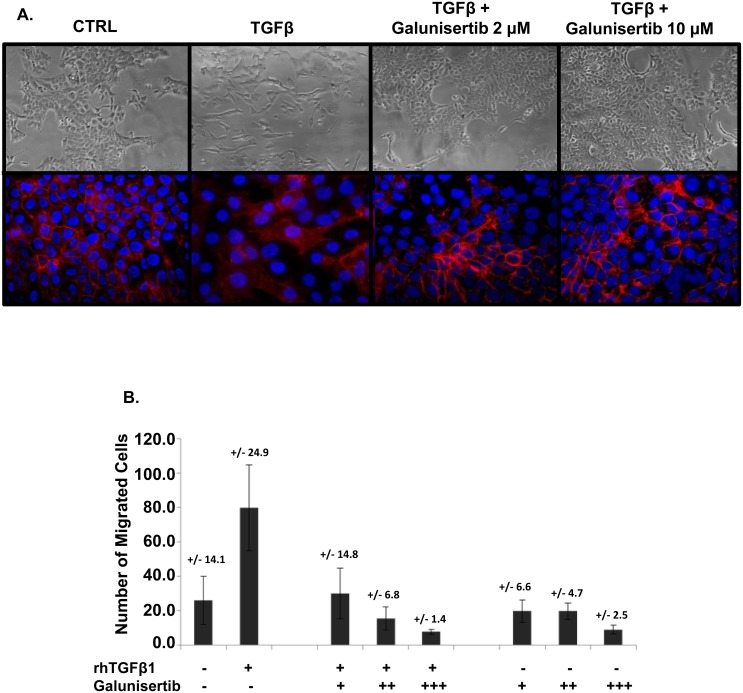
Galunisertib inhibits TGFβ mediated EMT and migration *in vitro* **A**. KPC-M09 cells were treated with control, TGFβ or TGFβ plus galunisertib (2 μM or 10 μM). Bright field images are shown in the top panel and were imaged at 20X. The bottom panel shows fluorescent IHC using anti-E-cadherin (red) as well as DAPI (blue) as a counter stain. Cells were imaged at 40X. **B**. U87MG cells were treated with control media (−), TGFβ alone (+, recombinant human, “rh”), TGFβ plus an increasing amount of galunisertib (+ = 1 μM, ++ = 3 μM, +++ = 10 μM), or galunisertib alone as indicated.

TGFβ is a well-characterized promoter of epithelial to mesenchymal transition (EMT) of tumor cells, a process associated with expression of a more mesenchymal cell phenotype and enhanced migratory properties of cells, ultimately leading to increased malignant potential [7]. EMT is well described to be a key process leading to more aggressive disease. To evaluate the ability of galunisertib to inhibit TGFβ1-mediated EMT, a TGFβ-dependent model system was employed using the mouse pancreatic cancer cell line KPC-M09. In the absence of TGFβ1 treatment, KPC-M09 cells showed membrane expression of the epithelial cell marker E-cadherin (Figure 5A, bottom panel). Bright field images show an epithelial morphology (Figure 5A, top panel). Treatment with TGFβ1 resulted in EMT as demonstrated by loss of epithelial cell morphology and absence of expression of E-cadherin (Figure 5A). Addition of galunisertib prevented TGFβ1-mediated EMT, as cells remained epithelial in phenotype and continued to express membrane E-cadherin (Figure 5A).

To evaluate the ability of galunisertib to inhibit TGFβ1-mediated cell migration, human U87MG glioblastoma cells were utilized in an *in vitro* migration assay. Treatment of U87MG cells with TGFβ1 enhanced migration of U87MG cells. Addition of galunisertib blocked cell migration in a dose dependent manner (Figure 5B). Notably, in this model system, galunisertib reduced baseline migration of U87MG cells in the absence of exogenous TGFβ1, presumably by inhibiting autocrine signaling through TGFβRI.

### Galunisertib reverses TGFβ1-mediated immune suppression of CD8^+^ T cells and NK cells

**Figure 6:**
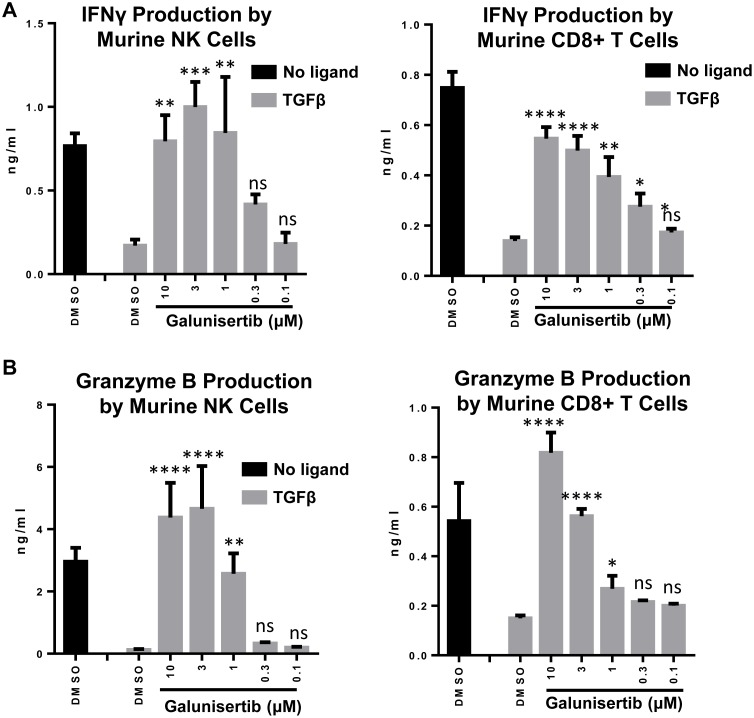

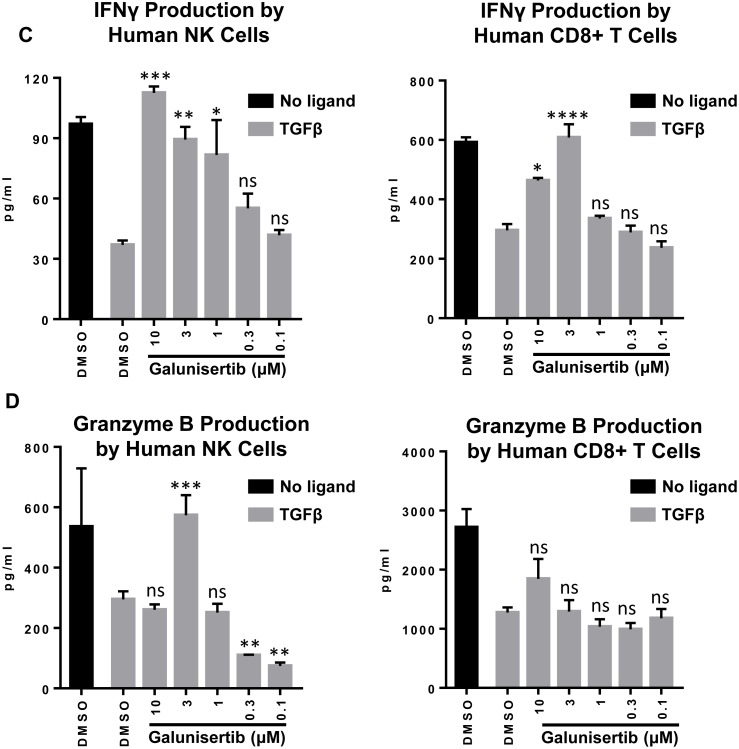
Galunisertib inhibits TGFβ mediated immune suppression of anti-tumor cytokines *in vitro* **A.**, **B.** Murine NK and CD8+ T cells were isolated from BALB/c mice and treated with vehicle alone, TGFβ or TGFβ plus galunisertib. **C.**, **D.** Human NK and CD8^+^ T cells isolated from PBMCs of healthy donors were treated and analyzed in a similar fashion. IFNγ (A, C) and granzyme B (B, D) in supernatants were analyzed by ELISA. Representative examples are shown. One-way ANOVA with Dunnett's test was used to compare the TGFβ+galunisertib treatments to the TGFβ+DMSO treatment. ****: *p* ≤ 0.0001; ***: *p* ≤ 0.001; **: *p* ≤ 0.01; *: *p* ≤ 0.05; ns: *p* ≥ 0.05

TGFβ1 can inhibit effector functionality in lymphocyte subsets. To evaluate the ability of galunisertib to rescue immune effector functions in the presence of TGFβ1 we tested murine and human lymphocytes. Freshly isolated murine or human NK or CD8^+^ T cells were stimulated with CD3/CD28/CD2 beads, treated with TGFβ1, with or without galunisertib. In the absence of galunisertib, TGFβ1 potently inhibited secretion of IFNγ (Figure 6A, 6C) and granzyme B (Figure 6B, 6D) by murine and human primary NK and CD8^+^ T cells. Addition of galunisertib to the TGFβ1 treated cells prevented the suppression of IFNγ and granzyme B secretion in murine as well as human NK and CD8^+^ T cells in a dose-dependent manner. Notably, in the absence of TGFβ1 ligand, galunisertib treatment appeared to result in enhanced granzyme B or IFNγ production by NK and CD8^+^ T cells in certain instances, potentially reflecting a role for autocrine TGFβ inhibition in the production of effector molecules by these cell subsets.

### Antitumor efficacy of galunisertib *in vivo*

**Figure 7:**
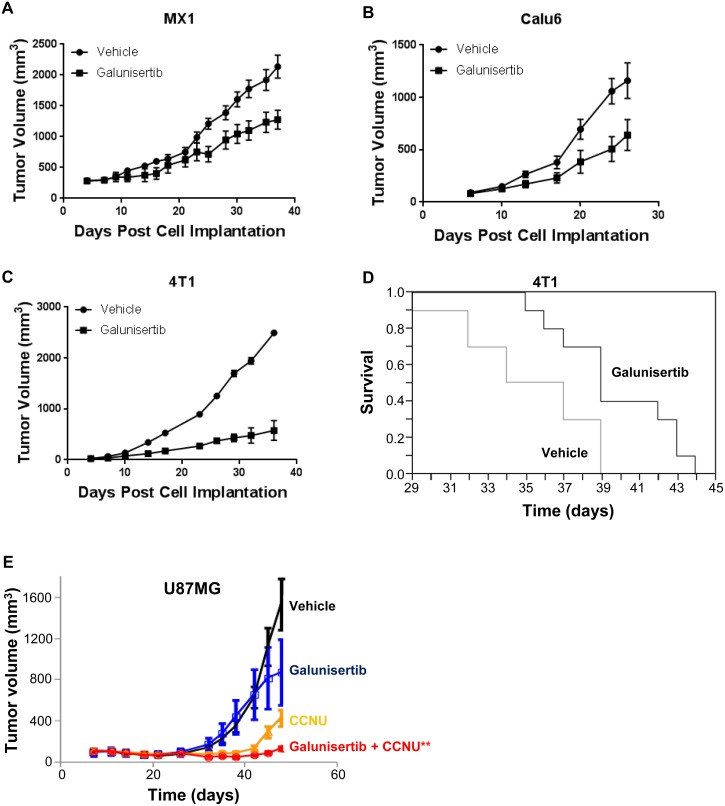
Galunisertib inhibits tumor cell growth *In vivo*, in both immune compromised and immune competent tumor models **A.**, **B.** Change in tumor volume in galunisertib or vehicle treated (75 mg/kg BID) MX1 (A, *p* = 0.014) or Calu6 (B, *p* = 0.034) human xenograft tumors. These data were presented in a previous publication [31]; however, the tumor growth delay observed was not reported. **C.** Tumor volume measured in galunisertib treated (75 mg/kg BID) 4T1 syngenic breast cancer model. *P* < 0.01. **D.** Kaplan-Meier analysis of survival in 4T1 syngenic breast cancer model after vehicle (gray line) or 75 mg/kg twice daily galunisertib, black line) treatment. *P* = 0.01. **E.** Tumor volume change in the U87MG xenograft model following treatment with vehicle, galunisertib (25 mg/kg), CCNU (lomustine, 30 mg/kg) or a combination of galunisertib and CCNU. *p* < 0.05, **p < 0.001 *versus* all other dose groups.

The *in vivo* antitumor efficacy of galunisertib was evaluated using a dose of 75 mg/kg administered twice daily by oral gavage, the dosing schedule defined by the PK/PD profile described in the pSMAD inhibition assays (Figure 4). Monotherapy anti-tumor activity of galunisertib was evaluated in three independent models; the immune competent 4T1 syngenic murine breast cancer model, the MX1 human xenograft breast cancer model, and the Calu6 human xenograft lung cancer model. In each of these established tumor models, galunisertib monotherapy resulted in significant tumor growth delay (Figure 7A, 7B, 7C, and 7D). For MX1, galunisertib monotherapy resulted in tumor growth delay of 10.3±4.3 days (1500 mm^3^ crossing time, *p* = 0.014) (Figure 7A) and for Calu6 galunisertib monotherapy resulted in tumor growth delay of 8.3 +/− 2.6 days (500 mm^3^ crossing time, *p* = 0.034) (Figure 7B); for 4T1, galunisertib monotherapy resulted in a tumor growth delay of 13±2.4 days (500 mm^3^ crossing time, *p* < 0.01 by repeated measures analysis) and a survival advantage of 4.5 days (*p* = 0.01) (Figure 7C, 7D), demonstrating the antitumor activity of the compound in traditional preclinical tumor models. No body weight loss was observed in these models (data not shown).

The ability of galunisertib to combine with chemotherapeutic agents and inhibit tumor growth was evaluated in a glioblastoma model. In these studies, galunisertib was administered to immune compromised animals implanted with U87MG tumor cells, alone or in combination with lomustine (CCNU), which is used clinically to treat glioblastoma (Figure 7E). In this model, galunisertib monotherapy had a modest anti-tumor effect; lomustine also had antitumor activity as monotherapy; however, combination of galunisertib and lomustine resulted in a significant reduction in tumor volume compared to all other treatment groups.

### Correlation between tumor and PBMC target modulation in the 13762 rat mammary carcinoma model

**Figure 8:**
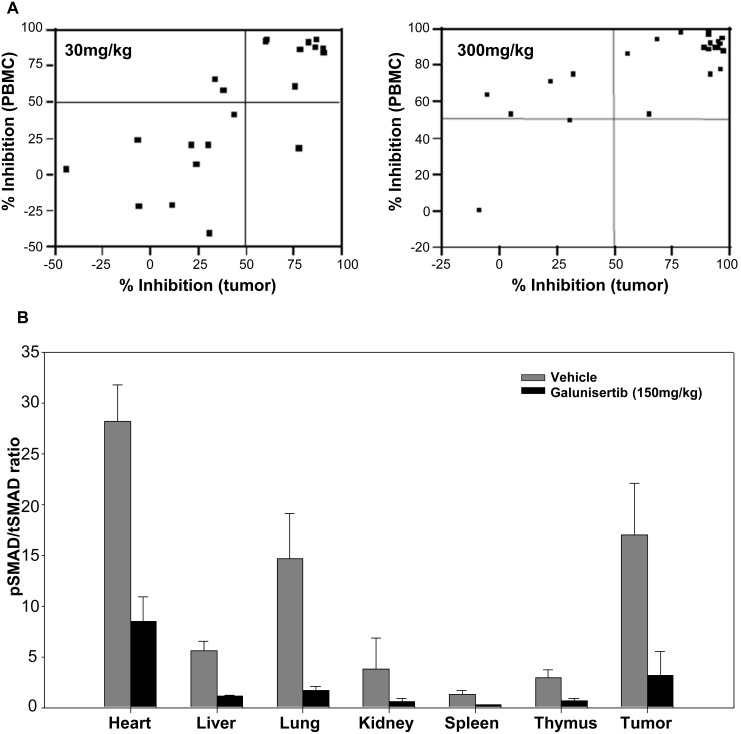
PBMCs as a surrogate tissue for assessing pSMAD modulation in tumors **A.** Correlation between tumor and PBMC target modulation in the 13762 rat mammary carcinoma model after a 30-mg/kg or 300-mg/kg dose. **B.** Ratio of relative pSMAD/tSMAD protein levels in vehicle- and galunisertib-treated rat tissues following a single dose of galunisertib at 150mg/kg. The pSMAD/tSMAD ratio was calculated from relative pSMAD and tSMAD levels in each tissue, as described under statistical analyses in the Methods section. Percent inhibition values were compared between treated and vehicle controls.

Galunisertib has been shown to inhibit pSMAD in human PBMCs *ex vivo* [32]. To evaluate the potential to use PBMCs to study the pharmacodynamic properties of galunisertib *in vivo*, we utilized a rat model and assessed the degree of pSMAD inhibition in PBMC, tumor tissue, as well as a range of normal tissues. A single oral administration of galunisertib at two different doses (30 and 300 mg/kg), resulted in a significant linear correlation between percent pSMAD inhibition in tumor tissue and PBMC (0.75 for 30 mg/kg, 0.78 for 300 mg/kg, and 0.75 for the combined data, Figure 8A). The IC_50_ of pSMAD in tumor tissue was estimated to be 0.719 μM (with a CV of 25.7%), and 1.96 μM (with a CV of 13.1%) in PBMC (Table 4). The I_max_ values of pSMAD in tumor and PBMCs were estimated to be 86% (with a CV of 3.4%) and 94.7% (with a CV of 1.1%), respectively (Table 4). A single oral administration of galunisertib at 150 mg/kg similarly resulted in inhibition of pSMAD in a variety of normal tissues (Figure 8B). These results were consistent with target modulation in *ex vivo* human PBMCs that has been reported [32]. Overall, there was a marked decrease in pSMAD/tSMAD ratio in galunisertib-treated rat tissues as compared to vehicle-treated rats (Figure 8A and 8B).

**Table 4:** PD parameters of phospho-SMAD in tumor and PBMC

Parameters and %CV	Tumor	PBMC
I_MAX_ (%)	86 (3.4%)	94.7 (1.1%)
IC_50_ (μmol/L)	0.719 (25.7%)	1.96 (13.1%)
K_OUT_ (H-^1^)	36.7 (7.3%)	47.4 (1.8%)

Abbreviations: CV = coefficient of variation, IC_50_ = inhibitory concentration of 50%, I_MAX_ = maximal inhibition, K_OUT_ = first order rate constant, PBMC = peripheral blood mononuclear cells, PD = pharmacodynamic

## DISCUSSION

The TGFβ pathway has diverse and important functions in the malignant process, including enhancement of tumor cell proliferation, induction of a more aggressive tumor phenotype through EMT, and suppression of the anti-tumor immune response. Accordingly, targeting the TGFβ signaling pathway has been an attractive objective for cancer therapy, and several molecules that inhibit the TGFβ pathway are under clinical development [21, 24, 33]. Here we describe the structural and functional properties of galunisertib, a potent, selective inhibitor of TGFβRI kinase activity. We show that galunisertib has selectivity for the TGFβ pathway, and potently inhibits TGFβ-mediated phosphorylation of SMAD2/3. We further show that galunisertib treatment inhibits TGFβ1-driven EMT and migration of tumor cells, reverses TGFβ1-mediated immune suppression, and inhibits *in vivo* growth of human tumor xenografts and syngenic murine tumors. Finally, we provide data to demonstrate that tissue pharmacodynamic properties of galunisertib can be effectively monitored in peripheral blood. Together, these data support the continued clinical development of galunisertib to target tumors dependent on TGFβ-driven biology for growth, metastasis, and immune evasion.

Galunisertib continues to advance in clinical trials. The compound has completed Phase I [34] and is currently under investigation in several Phase II trials. The initial phase I trial showed promising activity in patients with glioblastoma [34]. In phase II trials galunisertib failed to show improvement over lomustine in second line glioblastoma [35]; however, it did show clinical activity in patients with myelodysplastic syndrome [36] and superiority over gemcitabine in patients with pancreas cancer [37]. Thus far, galunisertib has been well-tolerated as a first-in-class, oral cancer therapy, and remains a promising compound in clinical development [19, 21].

The data presented in this report expand on the pharmacodynamic modeling of galunisertib in the Calu6 human model [31], demonstrate a robust PK/PD relationship across different species including mice and Fischer 344 rats, and establish optimal dosing and scheduling parameters to support maximal target modulation *in vivo*. Using an optimized dose and schedule, galunisertib demonstrated potent antitumor efficacy in multiple *in vivo* models, including human tumor xenografts and 4T1 syngenic mammary tumors. Our results are consistent with and expand the impact of prior reports that showed systemic treatment with monoclonal antibodies that target TGFβ ligands or the type II TGFβ receptor inhibit metastatic invasion of breast cancer cells in the 4T1 model [38–39], and previous reports on the impact of systemic treatment with small molecule inhibitors of TGFβ on *in vivo* xenograft and murine models of metastatic pancreatic cancer and glioblastoma [16–18, 40]. In addition, our data further support prior reports showing the activity of the TGFβ pathway in promoting MDS [41]. Based on this information, a pre-defined PK/PD model guided the first-in-human dose escalation study and was critical for defining a predictive and safe therapeutic window [34].

Beyond its key role to support the malignant phenotype, TGFβ is also pivotal for embryonic development, tissue homeostasis, and wound healing [4, 42]. In fact, TGFβRI kinase (Alk5) inhibitors, AZ12601011 and AZ1279934, induced cardiac valve lesions in rats [43]. Accordingly, we observed inhibition of pSMAD in a variety of normal tissues following administration of galunisertib to rats. Thus, inhibiting TGFβ signaling in non-target tissues may lead to adverse reactions. In corroboration with these earlier reports, high doses of galunisertib were associated with adverse effects in rats; these data have been presented elsewhere [44]. Thus high stringency must be adopted in the selection of dosing regimens in human subjects to achieve the desired effect with minimal toxicity. The ability to accurately assess the PK/PD profile of galunisertib based on pSMAD inhibition in PBMC provides a relatively non-invasive method to establish PK/PD relationships for galunisertib in patients with cancer. The ability to make such an assessment is likely to be of significant importance across molecules that target the TGFβ pathway.

Our results provide further evidence of the therapeutic value of inhibition of the TGFβ pathway in cancer and provide mechanistic rationale supporting further investigation of galunisertib.

## MATERIALS AND METHODS

### Crystallography and selectivity profiling of galunisertib

TFGβRI (T204D) was expressed in Baculovirus. The cells were harvested by centrifugation and re-suspended in lysis buffer (10 mM Tris-HCl pH 7.5/25 mM NaCl/5 mM MgCl_2_, 0.25 mg/ml PMSF/5 μg/ml Leupeptin/10 μg/ml Aprotinin and 0.5 mM ATP). Then cells were homogenized and disrupted by Kimax^®^ homogenizer. Insoluble material was removed by centrifugation and the supernatant was passed through a Q Sepharose FF column. The flow through was passed through HisTrap FF column. The bound protein was eluted followed by subsequent TEV digestion. The digested sample was purified by second HisTrap FF and Superdex200^®^ columns.

Protein was concentrated to 5 mg/ml in buffer consisting of 10 mM Tris pH7.6, 10 mM MgCl_2_, 2 mM ATP, and 3% 1,6-hexanediol (Hampton Research). TGFβRI was crystallized using vapor diffusion method. Crystals were grown at 20°C in hanging drops by mixing 1 μl of protein and 1μl of a reservoir solution (Hampton Research Crystal Screen #17 with 0.2 M Lithium sulfate monohydrate/0.1 M TRIS pH 8.5/30% w/v Polyethylene glycol 4,000). Crystals were then transferred to a soaking solution containing 2 mM ligand for 48 hours. Harvested crystals were quickly transferred to reservoir solution supplemented with 20% glycerol before being flash-frozen in liquid nitrogen.

KINOMEscan^®^ *in vitro* kinase competition binding assays (DiscoverX) were used to compare and quantitatively measure interactions between galunisertib and 456 kinases. Compound was provided to the vendor as 10 mM stocks in dimethylsulfoxide (DMSO). Galunisertib was diluted to create a 3 point concentration-response curve (CRC, concentration 20 μM, 2 μM, and 0.2 μM in 4% final DMSO concentration). Compounds, which bind the active site of the enzyme, prevent kinase binding to the immobilized ligand. Compounds that do not bind the active site of the enzyme, do not impact enzyme binding to the immobilized ligand. Thus, compound sensitivity is monitored by measuring the amount of kinase captured in test versus control samples by using qPCR. Binding affinity for test compound-kinase interactions was calculated by measuring the amount of kinase captured on the solid support as a function of the test compound concentration. Non-standard IC_50_ values were obtained from 3-point dose responses. Data are presented as Geomean ± SD.

Galunisertib was also tested in duplicate at 10 μM for activity in the CEREP diversity panel against 65 targets (Upstate Biotechnology).

### TGFβRI-T204D Ki determination and kinase assays

Ki values and mechanism of action of galunisertib were determined by filter binding assay. Briefly, in a 96-well microplate, the titrations for an inhibitor, ATP, and substrate phospho-SMAD3 (pSMAD3) were prepared. A 6-point titration was utilized for galunisertib ATP and the substrate. ATP titration utilized concentrations of 0, 6.25, 12.5, 25, 50, and 100 μM and the substrate pKSMAD3 and hyperactive mutation T204D were fixed at 200 μM and 100 nM, respectively. pKSMAD3 substrate titration utilized concentrations of 0, 50, 100, 200, 400, and 800 μM and ATP and hyperactive mutation T204D were fixed at 25 μM and 100 nM, respectively. The reactions were incubated for 30 to 40 minutes at 30°C, and terminated by the addition of 0.5% phosphoric acid. The Ki and the competition mechanism of each inhibitor were determined by kinetic analyses with Mathematica^®^ (version 4.1) software.

### Cell-based reporter assays

Cell-based reporter assays were used to assess activity of galunisertib in activating SMAD2/3 activity or SMAD1/5/8 activity. Briefly, Human HEK293 cells were engineered to express the firefly luciferase reporter gene either from a SMAD2/3- responsive promoter in response to TGFβ1 ligand stimulation or from a SMAD1/5/8-responsive promoter (hepcidin) in response to the bone morphogenic protein 4 (BMP4) ligand. Plates with either the SMAD2/3 reporter line or the SMAD1/5/8 reporter line were treated with a titration of galunisertib then TGFβ1 (final concentration = 2 nM, R&D Systems) or BMP4 (final concentration = 20 nM, R&D Systems). Cells were then lysed and assayed for luciferase activity of the reporter gene [Promega Bright Glo Luciferase Reagent (Cat #E2620) and Glo Lysis Buffer (Cat #E2661)]. Percent inhibition of compound treated groups was calculated relative to the minimum inhibition group (DMSO alone, untreated). Relative IC_50_ for galunisertib was calculated from a dose response study and is the concentration necessary to achieve 50% inhibition. Autophosphorylation assays were employed using recombinant TGFβRI (T204D) or TGFβRII proteins as previously described [26]. Radioactively labeled ATP was used in the 60-minute enzymatic assay. After incubation on a filter plate, radioactive counts were collected and the IC_50_ determined.

### Mink lung epithelial cell p3TP-Lux reporter assay

The TGFβ-responsive p3TP-Lux reporter construct was stably expressed in Mv1Lu mink lung epithelial cells. Cells were plated at a density of 1.5×10^4^ cells/well in black isoplates (Wallac) and incubated overnight for adhering. Media was replaced with 0.5% FBS/DMEM and incubated at 37C/5% CO_2_ for 2 hours. Galunisertib was diluted in 0.5% FBS/DMEM containing 10% dimethyl sulfoxide (DMSO) and was added to cells and incubated at 37°C/5% CO_2_ for 2 hours. Cells were treated with either 0.5% FBS/DMEM alone or TGFβ1 diluted in 0.5% FBS/DMEM and incubated overnight (12 to 18 hours). The following day, cells were washed with PBS and lysed with lysis buffer by incubating for 20 to 30 minutes at room temperature. Counts were taken on the MicroBeta JET by injecting 50 μL Luciferase Assay Reagent II (PROMEGA), delay for 1 second and count for 5 seconds. Curve fitting utilized a 4-parameter logistic model and absolute IC_50_ values were reported.

### ^3^H-thymidine proliferation assay

NIH3T3 cells or Mv1Lu cells were plated at a density of 1.5 × 10^4^ cells/well in white isoplates (Wallac) and were allowed to adhere overnight. The following day, media was replaced with 70 μL of 0.5% FBS/DMEM. Galunisertib was diluted in 0.5% FBS/DMEM containing 10% DMSO and added to cells and incubated at 37°C/5% CO_2_ for 2 hours. Cells were treated with either 0.5% FBS/DMEM alone or TGFβ1 diluted in 0.5% FBS/DMEM and incubated overnight (12 to 18 hours) to allow cells to complete 1 cell cycle. 20 μL ^3^H-thymidine solution (25 μCi/mL final concentration) was added to each well and incubated for 6 hours at 37°C/5% CO_2_. Media was aspirated and cells were washed with 1x PBS. 200 μL Supermix scintillation cocktail (Wallac) was added and incubated for 1 hour to allow complete cell lysis. Counts were taken as described previously. For bFGF and PDGF proliferation assays, NIH3T3 cells were used and these growth factors replaced TGFβ1 in the above protocol.

### pSMAD inhibition by Western Blot or ELISA

For *in vivo* measurement of tumor cell pSMAD activity, tumors were harvested from mice at the indicated time points post-treatment, snap frozen in liquid nitrogen, then pulverized in lysis buffer [EDTA 5 mM, EGTA 5 mM, sodium pyrophosphate 30 mM, sodium chloride 6.7 mM, sodium fluoride 50 mM, and 1% triton in water, pH 7.4 with protease and phosphatase inhibitors (Sigma, p8340, p5726, p0044) and sodium orthovanadate 2.5 mM]. Protein concentration was estimated with DC protein assay kit (BioRad).

For *in vitro* measurements of pSMAD activity, the murine 4T1-LP (luciferase positive) or EMT6-LM2 (EMT6 lung metastasis, passage 2) cells were cultured in 6-well plates in complete media (RPMI with 10% FBS; 0.8 mg/mL G418 selection for 4T1-LP only) until 90% confluent then serum starved overnight in Opti-MEM. Tumor cells were then incubated for 1 hour with galunisertib (starting at 10 μM, reduced by two-fold dilutions to 0.20 μM) in duplicate. The final concentration of DMSO was maintained in each sample at 0.1%. After incubation with the inhibitor, TGFβ1 was added from a 1000-fold stock (10 μg/ml in 40 mM acetic acid, with 0.1% BSA, Invitrogen) to a final concentration of 10 ng/ml to each well, except for the no TGFβ1 control, and the cells were incubated for 45 minutes at 37°C. The reaction was stopped by adding ice cold PBS, then excess TGFβ1 was removed and lysis buffer was added. Next, the samples were homogenized for 15 seconds using Bioruptor^®^ UCD-200 by Diagenode at medium power. Protein concentration for each lysate was measured using the BCA protein assay kit (Pierce #23225), then protein concentration of the lysate was normalized to 10 mg/mL with lysis buffer.

### Western blot

Forty to 50 μg of protein per lane were loaded on 8% to 10% SDS-PAGE then transferred to nitrocellulose membrane for western analysis. Membranes were blocked in 5% blocking buffer for 15 to 30 minutes. Antibodies detecting total SMAD (tSMAD) or pSMAD were diluted in blocking buffer (1:2000 tSMAD, Millipore anti-SMAD2/3 #07-408; 1:500 pSMAD, anti-pSMAD2 antibody, #04-953). Membranes were incubated with the primary antibody at 4°C overnight with continuous shaking. Membranes were washed 2 to 3 times for 15 minutes in wash buffer and then incubated in secondary antibody (1:1000-1:2000 in the blocking buffer, Amersham anti-rabbit IgG; #NA-934V) for one to two hours with continuous shaking. Membranes were washed three times for >20 minutes in wash buffer. Signal was detected with Supersignal WestPico Luminol/enhance solution (#1856136 PIERCE) as directed in the package insert. Band intensity was digitally captured by Lumi-Imager (Roche). Curve fitting utilized a four-parameter logistic model and absolute IC_50_ values were reported.

### ELISA

Total and pSMAD2/3 levels were assessed independently on two separate ELISA plates. While the coating antibody (anti-SMAD2/3 monoclonal antibody, BD Biosciences #62-4097) was the same for both plates, the secondary antibody was specific for either tSMAD2/3 or pSMAD2. To the pSMAD ELISA plate, 100 μL per well of lysate (ranging from 0.04-0.1 mg total protein) was added to the appropriate wells. To the total SMAD ELISA plate, 10% of the total protein that was added to the pSMAD, was added to the appropriate wells (0.004-0.01 mg protein final). A standard curve was also added to both ELISA plates (p- and tSMAD plates) using recombinant HIS-pSMAD2 protein (Eli Lilly and Company). Plates were incubated overnight then washed four times with wash buffer. Secondary antibody, anti-pSMAD2 antibody (rabbit monoclonal antibody #04-953) or Millipore anti-SMAD2/3 (rabbit polyclonal antibody #07-408) at 1:500 dilution in lysis buffer was added to the appropriate plate. Plates were incubated at room temperature for 2-3 hours, washed four times with wash buffer and reporter antibody (anti-rabbit HRP, Millipore #12-348, diluted 1:10,000 in blocking buffer) was added to the plates. Following one hour of incubation, plates were washed and developed using 3, 3’, 5, 5’-tetramethylbenzidine substrate (TMB; Surmodics/BioFX #TMBW-0100-01) and absorbance (OD) was read at 450 nm on a plate reader.

### Normalization and analysis of percentage inhibition

Percent inhibition of normalized pSMAD2 levels was calculated by first determining the ratio of tSMAD to pSMAD values for the vehicle group. Values were first normalized using the formula: normalized pSMAD2 = pSMAD2 (ng/mL)/sqrt [tSMAD2 (ng/mL)] and then this result for vehicle group was used to establish the minimum inhibition (0%) of pSMAD signal. Percent inhibition for compound treated groups was determined relative to the minimum pSMAD inhibition of the vehicle group using the formula: [(100- (normalized pSMAD in the experimental treatment group/normalized pSMAD in the vehicle treated group)]. IC_50_ of pSMAD inhibition was calculated for the data using non-linear regression to fit the standard four parameter logistic model using GraphPad Prism 6.

### Calu6 and EMT6-LM2 *in vivo* target inhibition

Female athymic nu/nu mice or immune competent syngenic BALB/c mice (Charles River or Taconic Farms, respectively, 22 to 28 grams) were maintained in microisolator cages, received food and water ad libitum, and were quarantined for 1 week before experimental manipulation. Sub-confluent Calu6 human non-small cell lung cancer (NSCLC) cells were injected subcutaneously in the flank of nude mice (1x 10^7^cells/per animal) in 0.2 mL of culture medium mixed with Matrigel (BD Biosciences, 1:1 v/v). EMT6-LM2 murine triple negative breast cancer tumor cells (5 × 10^5^ cells/per animal in the absence of matrigel) were implanted on the flank of BALB/c mice. EMT6-LM2 cells were generated from parental EMT6 cells which were passaged twice from spontaneous lung metastases (LM) following subcutaneous growth. When the tumor volume reached ~250 mm^3^, mice were randomized and separated into treatment groups.

For dose-response assays, galunisertib was administered orally by gavage in either 10% beta-cyclodextrin-HCl or 1% carboxy methyl cellulose (CMC)/0.5% sodium lauryl sulfate (SLS)/ 0.085% povidone vehicle preparation (PVP)/anti-foam (AF) (CMC/SLS/PVP/AF) formulation buffer. Tumors and plasma were collected 2 hours post dosage for analysis of pSMAD and compound levels.

For time-course assays, galunisertib was administered orally by gavage in CMC/SLS/PVP/AF at a dose of 25 mg/kg (EMT6-LM2) or 75 mg/kg (Calu6). Tumors and plasma were collected at the indicated time points (0.5, 1, 1.5, 2, 4, 8, and 16 hours) for analysis of pSMAD and compound levels.

Plasma samples were stored at −80°C prior to LC/MS/MS analysis for concentrations of galunisertib. A four-parameter logistic model was applied to define the galunisertib dose-response and exposure-response relationships. Percent target inhibition was calculated as described above then was modeled as a function of either dose or plasma concentration.

### Cell migration

U87MG cells were cultured in minimum essential medium (MEM) supplemented with 10% fetal bovine serum (FBS). Eight micron migration plates (24 well) were coated with denatured collagen I (BD, cat# 354326) at 10 μg/ml. Following overnight incubation at 4°C, the lower chambers were washed three times with phosphate buffered saline (PBS) followed by blocking with 1% bovine serum albumin (BSA) for 1 hour. The chambers were washed 3 times in PBS then 200 μl of migration buffer [MEM containing 5% blocking solution (Sigma, cat# 2393), 100 mM MgCl_2_, and 100 mM MnCl_2_] was added. Next, 10,000 cells in 100 μl migration buffer were added to the upper chamber then treated with 100 μl of galunisertib (corresponding to 10 μM, 3 μM, or 1 μM) or control solution. After 1 hour of culture, 10 ng/ml of recombinant human (rh) TGFβ1 was added to upper chambers and cells were allowed to migrate in a 37°C incubator for 48 hours. The non-migrated cells were aspirated and wiped off the upper chamber with a cotton swab. The inserts were washed 3 times with PBS and the chambers transferred to new 48 well plates. The chambers were fixed with 500 μl of 10% neutral buffered formalin (NBF) for 20 minutes followed by three washes with PBS. The cells were stained with Hoechst at 1:10,000 for 20 minutes in the dark then washed three times with PBS. The membranes were cut with a scalpel, placed on a slide and the pictures were taken using Eclipse 90i Microscope. Image Pro-Analyzer 7.0 was used for analysis of migrated cells.

### E-Cadherin assay

The primary mouse pancreatic ductal adenocarcinoma (PDAC) cell line KPC-M09 was derived from a pancreatic GEMM [*Kras*LSL-G12D/+; *Trp53*LSL-R172H/+; *Ptf1a*Cre/+ (KPC)]. Cells were treated with control, 30 ng/ml TGFβ, 30 ng/ml TGFβ plus 2 μM Galunisertib, or 30 ng/ml TGFβ plus 10 μM Galunisertib for 48 hours. Cell morphological changes were imaged at 20X (bright field). Cells were stained with an anti-E-cadherin primary Ab (Cell Signaling, clone 24E10, diluted 1:200) followed with a secondary anti-rabbit Cy3 (Cell Signaling, diluted 1:500). DAPI was used as a counter stain and cells were imaged at 40X.

### TGFβ suppression of murine NK and CD8^+^ T cell cytokine secretion

Mouse NK cells and CD8^+^ T cells were purified from female BALB/C mouse splenocytes by magnetic antibody sorting (Miltenyi Biotec) following the manufacturer's instructions. After isolation, the cells were re-suspended separately in complete media (RPMI1640, 5% FBS, 10 mM HEPES, 1×NEAA, 1×L-Glu, sodium pyruvate, 1×Penn/Strep, 0.055 mM βME, 50 U/ml IL2) and plated in flat 96-well plates at 37°C for 5-7 days at a density of 5 × 10^4^/well, in triplicate, and stimulated with Treg Suppression Inspector beads (Miltenyi Biotec, Cat# 130-093-627). Cells were cultured with or without TGFβ1 at 10 ng/ml. Galunisertib was added at concentrations ranging from 0.1 μM to 10 μM with DMSO as vehicle control. Cytokine release in supernatants was measured by ELISA (R&D, mouse Granzyme B: DY1865; mouse INFγ:3321-1H-6). One-way ANOVA with Dunnett's test was used to compare the TGFβ+Gal treatments to the TGFβ+DMSO treatment.

### TGFβ1 suppression of human NK and CD8^+^ T cell cytokine secretion

Blood from healthy donors were obtained from the New York Blood Center. After isolation of PBMCs via high density gradient centrifugation with Ficoll-Paque, NK cells and CD8^+^ T cells were purified from PBMCs by magnetic antibody sorting (Miltenyi Biotec) following the manufacturer's instructions. Purified CD8^+^ T cells and NK cells were re-suspended separately in complete media (RPMI1640, 5% FBS, 10mM HEPES, 1×NEAA, 1×L-glu, sodium pyruvate, 1×penn/strep, 0.055 mM βME, 50 U/ml IL-2) and plated in 96-well plates at 37°C for 2 days at a density of 5 × 10^4^/well, in triplicates. CD8^+^ T cells were stimulated with human T cell activation/expansion beads (Miltenyi Biotec; 130-091-441), while NK cells were stimulated with the same beads plus 10 U/ml IL-2. Cells were cultured with or without TGFβ1 at 10 ng/ml. Galunisertib was added at various concentrations (0.1 M to 10 μM) with DMSO as vehicle control. Cytokine release in supernatants was measured by ELISA (R&D; human Granzyme B: DY2906; human INFγ: DY285-05). One-way ANOVA with Dunnett's test was used to compare the TGFβ+Gal treatments to the TGFβ+DMSO treatment.

### MX1 and Calu6 *in vivo* xenograft cancer models

Female athymic nude mice from Charles River weighing 22 to 26 grams were implanted subcutaneously in the flank with 1×10^7^ cells in a 1:1 (v/v) mixture in matrigel. Administration of galunisertib at 75 mg/kg BID (150 mg/kg/day) formulated in CMC/SLS/PVP/AF or vehicle alone were initiated 4 to 6 days after injection of the tumor cells and continued for 20 consecutive days with 10 animals per group. Tumor volumes and body weight were determined by electronic caliper every 2 to 3 days. Repeat measures analysis was utilized with log transformed data and the auto-regression (AR) method to determine the overall p-value for galunisertib growth curves relative to the vehicle control in both tumor models. Repeat measures analysis was utilized with log transformed data and the AR method to determine the overall p-value for galunisertib growth curves relative to the vehicle control in both tumor models.

### 4T1*in vivo* syngenic breast cancer model

Female BALB/c mice from Taconic Farms weighing 20 to 24 grams were acclimated for 1 week prior to study initiation, had ad libitum access to food and were shaved in the implant area prior to the orthotopic injection of 1 × 10^5^ 4T1 cells into the mammary fat pad in a volume of 20 μL media. Ten animals per group were utilized for tumor measurement and survival endpoints. Administration of 75 mg/kg of galunisertib orally twice daily formulated in CMC/SLS/PVP/AF vehicle or vehicle alone were initiated 4 days after injection of the tumor cells and continued until study termination. Tumor volumes were determined by electronic caliper every 2 to 3 days. Statistical analysis was performed by repeated measures analysis and Kaplan-Meier analysis.

### U87MG glioblastoma xenograft tumor model

Five million U87MG glioblastoma cells, obtained from ATCC (Manassas, VA), were injected subcutaneously in the flank of 8- to 9-week old, female, athymic nude mice (Harlan, Indianapolis, IN) in a 1:1 mixture of 1x PBS and matrigel (Becton Dickinson, Bedford, MA). Mice were monitored daily for palpable tumors. When tumors reached a group mean volume of 100 mm^3^, mice were randomized to treatment groups and treated with galunisertib in CMC/SLS/PVP/AF dosed orally by gavage (25 mg/kg BID) or vehicle alone [45]. Two hours after the ninth dose of galunisertib, mice bearing U87MG xenograft tumors were given a single intraperitoneal dose of lomustine (CCNU) (30 mg/kg). The tumor volumes were recorded at regular intervals following treatment.

### 13762 rat tumor model

Fischer 344 rats from Taconic Farms weighing 150 to 160 grams were acclimatized for 1 week prior to study initiation and had ad libitum access to food. Rats were shaved in the implant area then given a subcutaneous flank injection of 5 × 10^6^ 13762 rat mammary carcinoma cells in a 1:1 (v/v) mixture with Matrigel in media. Twelve days after inoculation, when tumor volumes were approximately 600 mm^3^, rats were dosed orally with 30 or 300 mg/kg galunisertib in a CMC/SLS/PVP/AF formulation. Tumor, PBMCs, and plasma were collected at 0.5, 1, 1.5, 2, 4, 8, 16, and 24 hours for further analysis. Plasma samples were stored at −80°C prior to LC/MS/MS analysis.

### *Ex vivo* PBMC stimulation in the 13762 model

*Ex vivo* stimulation and assessment of target modulation in PBMCs have been described in detail previously [31]. Briefly, whole blood (2 to 3 mL) was collected by cardiac puncture from Fischer 344 rats and transferred to BD Vacutainer CPT sodium citrate tubes. PBMCs isolated from whole blood were stimulated with TGFβ1 at a final concentration of 100 pM at 37°C for one hour. Cells were washed with cold PBS and lysed in 50-100 μL of lysis buffer, and pSMAD and tSMAD levels were assessed by immunoblot assay as described above.

### Statistical analyses

For correlation of pSMAD modulation in 13762 rat mammary carcinoma tumors and PBMCs, data were normalized using an analysis of co-variance of log pSMAD versus log tSMAD and treatment group. The slope (b) from this analysis was used to normalize the pSMAD values using pSMAD/(tSMAD)^b^. Percent inhibition was calculated by the following formula: 100-(y/x)*100 where y = normalized pSMAD value and x = mean normalized pSMAD in the vehicle control. Linear correlation was used to compare tumor and PBMC measurements. In addition, using a cutoff of 50%, a 2 × 2 contingency table analysis was performed using Fisher's exact test.

## Abbreviations

TGFβ: transforming growth factor β
TGFβRI: TGFβ receptor I
TGFβRII: TGFβ receptor II
EMT: epithelial-mesenchymal transition
t_max_: maximum time; C_max_
TED_50_: total effective dose at 50%
TEC_50_: total effective concentration at 50%
PK/PD: pharmacokinetic/pharmacodynamics
